# Meteorological Influences on Spatiotemporal Variation of PM_2.5_ Concentrations in Atmospheric Pollution Transmission Channel Cities of the Beijing–Tianjin–Hebei Region, China

**DOI:** 10.3390/ijerph19031607

**Published:** 2022-01-30

**Authors:** Suxian Wang, Jiangbo Gao, Linghui Guo, Xiaojun Nie, Xiangming Xiao

**Affiliations:** 1 College of Safety Science and Engineering, Henan Polytechnic University, Jiaozuo 454000, China; 2277410@163.com; 2 Key Laboratory of Land Surface Pattern and Simulation, Institute of Geographic Sciences and Natural Resources Research, Chinese Academy of Sciences, 11A Datun Rd., Beijing 100101, China; gaojiangbo@igsnrr.ac.cn; 3 School of Surveying and Land Information Engineering, Henan Polytechnic University, Jiaozuo 454000, China; niexj2005@126.com; 4 Department of Microbiology and Plant Biology, Center for Earth Observation and Modeling, University of Oklahoma, Norman, OK 73019, USA; xiangming.xiao@ou.edu

**Keywords:** PM_2.5_ concentration, spatiotemporal patterns, meteorological factors, “2 + 26” cities, Beijing–Tianjin–Hebei region

## Abstract

Understanding the spatiotemporal characteristics of PM_2.5_ concentrations and identifying their associated meteorological factors can provide useful insight for implementing air pollution interventions. In this study, we used daily air quality monitoring data for 28 air pollution transmission channel cities in the Beijing–Tianjin–Hebei region during 2014–2019 to quantify the relative contributions of meteorological factors on spatiotemporal variation in PM_2.5_ concentration by combining time series and spatial perspectives. The results show that annual mean PM_2.5_ concentration significantly decreased in 24 of the channel cities from 2014 to 2019, but they all still exceeded the Grade II Chinese Ambient Air Quality Standards (35 μg m^−3^) in 2019. PM_2.5_ concentrations exhibited clear spatial agglomeration in the most polluted season, and their spatial pattern changed slightly over time. Meteorological variables accounted for 31.96% of the temporal variation in PM_2.5_ concentration among the 28 cities during the study period, with minimum temperature and average relative humidity as the most critical factors. Spatially, atmospheric pressure and maximum temperature played a key role in the distribution of PM_2.5_ concentration in spring and summer, whereas the effect of sunshine hours increased greatly in autumn and winter. These findings highlight the importance of future clean air policy making, but also provide a theoretical support for precise forecasting and prevention of PM_2.5_ pollution.

## 1. Introduction

Particulate matter with an aerodynamic diameter of less than 2.5 µm (PM_2.5_) represents one of the most serious air pollutant types due to its profound effects on human living environments [1,2,3]. Previous studies have revealed that PM_2.5_ significantly affects atmospheric visibility through the scattering and absorption of solar radiation [4,5,6] and modification of cloud microphysical properties by acting as cloud condensation nuclei [7,8]. PM_2.5_ exerts extreme influences on human health and has been associated with respiratory problems, lung cancer, cardiovascular morbidity and infectious diseases [1,9,10]. Exposure to PM_2.5_ pollution was related to approximately 1.03 million deaths in the major cities of China in 2013 [11], 103.1 million disability-adjusted life-years and 4.2 million deaths worldwide in 2015 [12] and about 138,150, 80,945, and 18,752 premature deaths for all-cause, cardiovascular diseases and respiratory diseases, respectively, in the Beijing–Tianjin–Hebei (BTH) region in 2015 [13]. Therefore, quantifying the spatiotemporal variation of regional PM_2.5_ concentration and understanding its driving factors is essential for addressing global concerns in government, the scientific community and the general public.

The BTH region is the capital economic circle of China. Mainly due to the coal-based energy structure, road-dominated freight transport, fugitive dusts and agricultural activities, it has become one of the most severe air pollution regions in China [14,15,16]. To date, many studies have focused on the temporal and spatial variations of PM_2.5_ in this region. However, most of these studies have been limited to individual cities [17,18,19], dozens of major cities during certain seasons within short time series [20,21,22,23] or 13 cities within a single year [24,25]. Recently, some studies reported an obvious spatial spillover effect of air pollution among different cities [26,27], suggesting much larger amounts from surrounding cities. For example, Chang et al., 2019, indicated that, although local PM_2.5_ emissions were dominant in all 13 cities within the BTH area, regional transport from Shandong and Henan Provinces contributed as much as 12.9% of PM_2.5_ emissions [28]. To dramatically reduce the pollution in the BTH area, an air pollution prevention and control program for the BTH and surrounding areas including 28 cities was announced jointly by the Ministry of Ecology and the Environment of China in 2017. The regulations include mandating the shutdown of small and polluting factories, clean heating in winter by replacing coal with gas or electricity, staggered peak production in heating season and no open burning (Table S1). Thus, comprehensive and accurate investigation of the spatiotemporal variation of PM_2.5_ concentration in the 28 cities within the air pollution transmission channel of the BTH region is urgently needed as a scientific basis for preventing and controlling regional air pollution [29,30].

Meteorological conditions and synoptic circulation patterns exert important influences on PM_2.5_ variation [31,32]. Recently, a large body of research has been conducted based on the chemical transport models. Wang and Zhang, 2020, suggested that about a 59.9% decrease in PM_2.5_ concentration in 2017 was related to atmospheric conditions over the BTH region [33]. Gonzalez-Abraham et al., 2015, predicted that PM_2.5_ would increase by 10–30% across the eastern USA to the 2050s [34]. However, the accuracy of these model simulations is greatly influenced by the emissions inventory, model design and meteorological conditions [35]. Statistical models derived from observed PM_2.5_ and meteorological data offer an alternative approach to identify the effects of meteorological conditions on PM_2.5_ distribution [36]. However, due to strong relationships among meteorological factors, traditional statistical methods, such as correlation analysis and linear regression, tend to produce biased results when assessing the effects of individual meteorological variables on PM_2.5_ [37,38]. On the other hand, PM_2.5_ responses to meteorological variables are complex [31,39]. PM_2.5_ concentration could rise in areas where the temperature changed from comfortable to cold due to household heating [27] and temperature inversion [38]. It also could be positively related to daily temperature, especially in spring and winter, probably by decreased wind speed through the transformation of air pressure [14] and chemical reactions. The response of PM_2.5_ concentrations to changes in meteorological conditions seems to be heavily dependent on the time period, case terrain, atmospheric circulation and existing large spatiotemporal heterogeneities [40,41]. Therefore, analyses of the relationship between PM_2.5_ and meteorological variables should be conducted by combining time series and spatial perspectives to better quantify the role of meteorological factors in spatiotemporal PM_2.5_ variation. In February 2012, the new Chinese Ambient Air Quality Standards (CAAQS, GB3095-2012) were jointly issued by the Ministry of Environmental Protection and the General Administration of Quality Supervision, Inspection and Quarantine of the People’s Republic of China. It was not until the air quality standard was published that real-time hourly PM_2.5_ and PM_10_ concentration data for the major cities were collected and released online, which is essential for providing detailed information about particulate matter pollution and helpful for understanding how local air quality has changed over time.

In this study, we examined reductions in PM_2.5_ concentration during 2014–2019 in the 28 cities of the air pollution transmission channel in the BTH region of China at seasonal and annual scales. Then we explored the spatial heterogeneity of the relationship between PM_2.5_ concentration and meteorological variables based on geographical weight regression (GWR) analysis. Finally, we performed partial correlation and multiple linear regression (MLR) analyses to quantify PM_2.5_ concentration response to meteorological variables and their contributions to the PM_2.5_ concentration variation from a time-series perspective. The findings of this study will contribute to a more comprehensive understanding of the influence of meteorological variables on PM_2.5_ concentration and will provide strong theoretical support for precise forecasting and prevention of PM_2.5_ pollution in the BTH region.

## 2. Materials and Methods

### 2.1. Study Area

**Figure 1:**
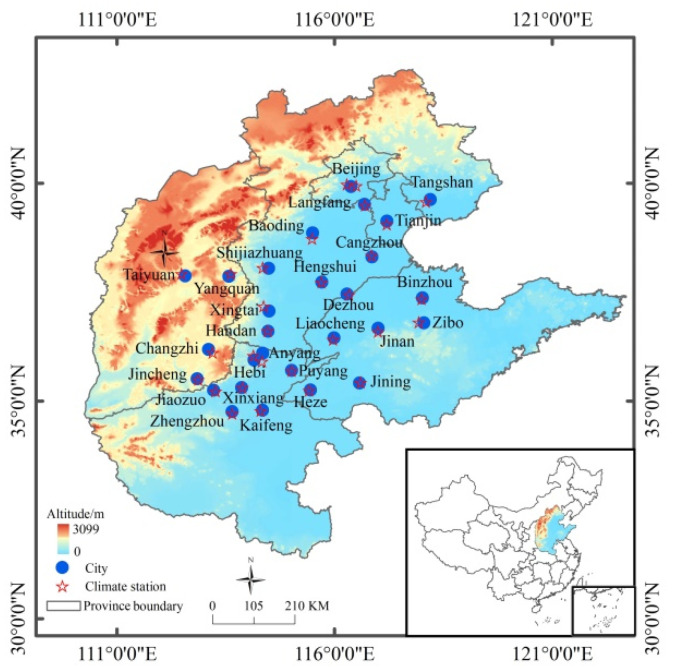
Locations of the 28 cities comprising the air pollution transmission channel in the Beijing–Tianjin–Hebei (BTH) area and regional meteorological stations.

The study area includes 28 air pollution transmission channel cities of the BTH region, covering an area of 275,000 km^2^ [30,39]. The 28 cities are the two municipalities of Beijing and Tianjin and 26 prefecture-level cities which comprise the air pollution transmission channel of the BTH region; these cities include Shijiazhuang, Tangshan, Langfang, Baoding, Cangzhou, Hengshui, Xingtai and Handan in Hebei Province; Taiyuan, Yangquan, Changzhi and Jincheng in Shanxi Province; Zhengzhou, Kaifeng, Anyang, Hebi, Puyang, Xinxiang and Jiaozou in Henan Province; and Jinan, Zibo, Jining, Dezhou, Liaocheng, Binzhou and Heze in Shandong Province (Figure 1). The area has a typical temperate monsoonal climate, with an annual mean air temperature of 4~15 °C and annual precipitation of 450~700 mm. The terrain is generally lower from west to east. Out of the 28 cities, 24 are located on the northern China plain, with altitudes below 200 m, whereas Taiyuan, Yangquan, Changzhi and Jincheng belong to the Loess Plateau, on the west side of the Taihang Mountains.

### 2.2. Data Sources

Daily PM_10_ and PM_2.5_ concentration data (μgm^−3^) between 1 March 2014 and 28 February 2019 for the 28 cities in the air pollution transmission channel of the BTH region (Jincheng data started from 1 March 2015) were obtained from an online database (https://www.aqistudy.cn/historydata, accessed on 8 December 2021). This dataset was constructed by averaging hourly concentration data for all national air quality monitoring sites within each city listed on the official website of the China National Environmental Monitoring Center [37]. In accordance with the China Environmental Protection Standards, each national air quality monitoring site recorded the real-time hourly PM_2.5_ and PM_10_ concentration obtained by the continuous automated monitoring system based on the micro-oscillating balance method and the β absorption method. The dataset contained very few missing records (44 days among all monitoring stations) and very few zero values for PM_10_ concentration. We interpolated missing values from the mean values of the two closest days to maintain time series data continuity and comparability. In addition, although PM_2.5_ has been paid special concern, it was not systematically observed and reported for most cities in China until 2014. Meanwhile, it was unreasonable to effectively consider the relative contributions of different particles to local air quality and the composition characteristics of particulate matter based only on fine particles (and without consideration of coarse particles). Therefore, we removed zero values of PM_10_ concentration and the corresponding PM_2.5_ concentration values from the time series in order to calculate the PM_2.5_/PM_10_ ratio, which provided crucial information related to particle origin and allowed retrospective prediction of PM_2.5_ concentration without direct PM_2.5_ measurements.

Daily meteorological data, including daily maximum temperature (*T_max_*, °C) and minimum temperature (*T_min_*, °C), average atmospheric pressure (*AP*, hPa), sunshine hours (*SH*, h), average relative humidity (*H*, %), maximum wind speed (*WS*, m/s) and wind direction (*WD*) for the 28 meteorological stations were collected from a Chinese meteorological data sharing service system (http://data.cma.cn/, accessed on 8 December 2021). The 28 meteorological stations were generally located at the center of each city (Figure 1). We interpolated missing values from the mean values of the two closest days and removed corresponding values of meteorological factors to square with records of PM_2.5_ concentration values from the time series.

### 2.3. Methods

#### 2.3.1. Spatial Autocorrelation and Linear Regression

The variation in PM_2.5_ concentration in each of the air pollution transport channel cities was investigated at seasonal and annual scales. In this study, spring, summer, autumn and winter were defined as March–May, June–August, September–November and December–February, respectively. Linear regression analysis was performed to examine the variation in PM_2.5_ concentration for each city. We also performed spatial autocorrelation analysis to evaluate the spatial associations of PM_2.5_ concentration by calculating the global and local Moran’s I using ArcGIS 10.4.1 software [26,42]. The global spatial autocorrelation describes the spatial dependence of PM_2.5_ in the whole area of the BTH region, which can be characterized by *Global Moran’s I*. The formula is as follows:

(1) $$\mathrm{Global}\mathrm{Moran}’s\;I=\frac{n\;\sum_{i=1}^{n}\sum_{j=1}^{n}W_{ij}\left(x_{i}-\bar{x}\right)\left(x_{j}-\bar{x}\right)}{(\sum_{i=1}^{n}\sum_{j=1}^{n}W_{ij})\sum_{i=1}^{n}{\left(x_{i}-\bar{x}\right)}^{2}}\;(i\;\neq j)$$

where *x_i_*, *x_j_*, are the PM_2.5_ concentration observations of cities *i*, *j*; *n* is the number of all cities; $\bar{x}$ is the average PM_2.5_ concentrations of all regions; *W_ij_* is the spatial weight matrix. *Global Moran’s I* values < 0, >0 and =0, indicate negative, positive, and random spatial correlation, respectively. Local Moran’s I values denote the local spatial autocorrelation patterns, where High–High (H–H) means that the city and surrounding cities have high PM_2.5_ concentrations; High–Low (H–L) represents that the city has a high PM_2.5_ concentration, but those of surrounding cities are low; Low–High (L–H) shows that the city has a low PM_2.5_ concentration, but those surrounding cities are high; Low–Low (L–L) means that both the city and surrounding cities have low PM_2.5_ concentrations; Not significant (NS) indicates no significant difference in PM_2.5_ concentration among cities. Global Moran’s I and Local Moran’s I statistical tests used a z-test at 95% confidence level. Finally, the coefficient of variation (CV) was calculated to investigate fluctuation in the temporal variability of PM_2.5_ concentration. We also explored the spatiotemporal variation in the PM_2.5_/PM_10_ ratios using the same methods.

#### 2.3.2. Partial Correlation and Multiple Linear Regression

Partial correlation analysis was performed to determine the relationships among temporal variations in daily PM_2.5_ concentration and meteorological factors for each city at seasonal and annual scales; this method has been successfully applied in previous environmental studies to remove covariate effects among multiple influential factors. Next, we performed multiple linear regression analysis of daily PM_2.5_ concentration and meteorological factors at seasonal and annual scales for each city, and the explanation ability of meteorological factors on PM_2.5_ variability was evaluated using the coefficient of determination (R^2^) [32,43]. All calculations were performed using MATLAB 2015b software, and statistical significance was determined at the level of *p* < 0.05.

#### 2.3.3. Geographically Weighted Regression

The geographically weighted regression was developed to deal with this non-stationarity, which allows relationships between independent and dependent variables to vary spatially by producing a set of local parameters to reveal spatial relationships by assuming heterogeneous influence of the same factor on dependent variables in different spatial units [44,45]. We performed geographically weighted regression to evaluate the spatial heterogeneity of the influence of meteorological factors on PM_2.5_ concentration based on site data for the 28 cities. First, we normalized mean seasonal and annual PM_2.5_ concentration and meteorological variable data during 2014–2019 for each city; we then conducted the geographically weighted regression analysis using the ArcGIS 10.4.1 software. The geographically weighted regression was run for an observation point by using a spatial kernel that centered on the point and weighted other observation points by a distance decay function. In this study, the Gaussian model was selected as a weighting function, and the corrected Akaike’s information criteria (AICc) was used to obtain the optimal bandwidth.

## 3. Results

### 3.1. Overview of PM_2.5_ Pollution

#### 3.1.1. Spatiotemporal Variation of PM_2.5_

**Figure 2:**
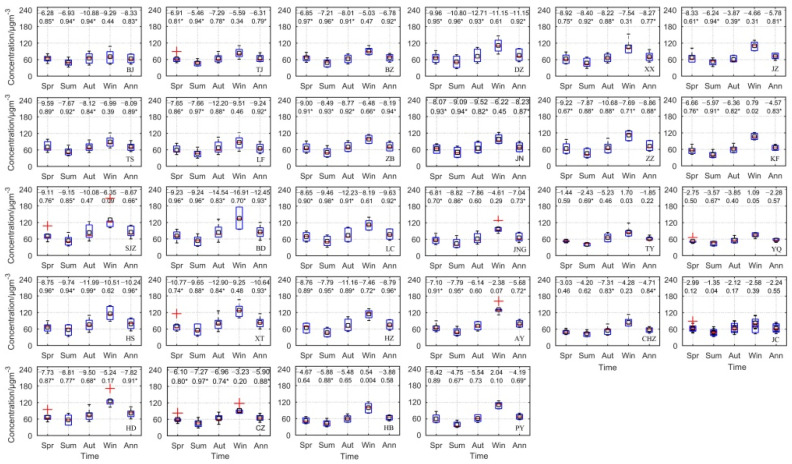
The temporal variation of mean PM_2.5_ concentration in the 28 air transport channel cities during 2014–2019. In each box plot, the bottom and top of the box mean the 25th and 75th percentiles, respectively; horizontal red lines indicate median values; open black squares represent mean values; whiskers indicate 1.5 times the interquartile range; and red crosses represent outliers. Linear regression slopes and *R*^2^ values are provided for each city (* *p* < 0.05): BJ, Beijing; TJ, Tianjin; SJZ, Shijiazhuang; TS, Tangshan; LF, Langfang; BD, Baoding; CZ, Cangzhou; HS, Hengshui; XT, Xingtai; HD, Handan; BZ, Binzhou; DZ, Dezhou; ZB, Zibo; JN, Jinan; LC, Liaocheng; JNG, Jining; HZ, Heze; AY, Anyang; HB, Hebi; PY, Puyang; XX, Xinxiang; JZ, Jiaozou; ZZ, Zhengzhou; KF, Kaifeng; TY, Taiyuan; YQ, Yangquan; CHZ, Changzhi; JC, Jincheng.

During 2014–2019, the highest annual mean PM_2.5_ concentrations were observed in Xingtai (83.69 μg m^−3^), Handan (80.98 μg m^−3^), Shijiazhuang (84.16 μg m^−3^) and Baoding (85.35 μg m^−3^), and the lowest PM_2.5_ concentration was observed in Shanxi Province, with a minimum of 56.77 μg m^−3^ in Yangquan (Figure 2). In all cities, the annual mean PM_2.5_ concentration exceeded the CAAQS (GB3095-2012) Grade II standard of 35 μg m^−3^. Mean PM_2.5_ concentration showed wide seasonal variation that was similar among all cities. The highest pollution levels were observed in winter and the lowest values in summer. However, the seasonal fluctuation range seriously differed among cities. For instance, Beijing, Tianjin, Tangshan, Langfang, Binzhou, Zibo and Yangquan showed lower CV values, whereas Baoding, Anyang, Puyang and Kaifeng had larger CV values (>40%). CV values were higher in winter and lower in spring. From 2014 to 2019, significant reductions in annual mean PM_2.5_ concentration occurred across 24 of the 28 channel cities (excluding Hebi, Taiyuan, Yangquan and Jincheng), with a mean rate of −7.14 μg m^−3^ year^−1^ (Figure 2). About half of the 28 cities showed a decrease rate of more than −8 μg m^−3^ year^−1^. These were mainly scattered in the Hebei and Shandong Provinces. However, in 2019, PM_2.5_ concentration exceeded the CAAQS (GB3095-2012) Grade II standard in all cities, and PM_2.5_ concentration variation showed significant seasonal and spatial distribution differences among all 28 cities. Compared with other seasons, PM_2.5_ concentration in spring and summer exhibited the clearest obviously decreasing trend, with significant reductions in 22 and 26 cities, respectively, which were mainly located in the Hebei and Shandong Provinces. Significant downward trends were also observed in 20 cities in autumn and only 4 cities in winter.

**Figure 3:**
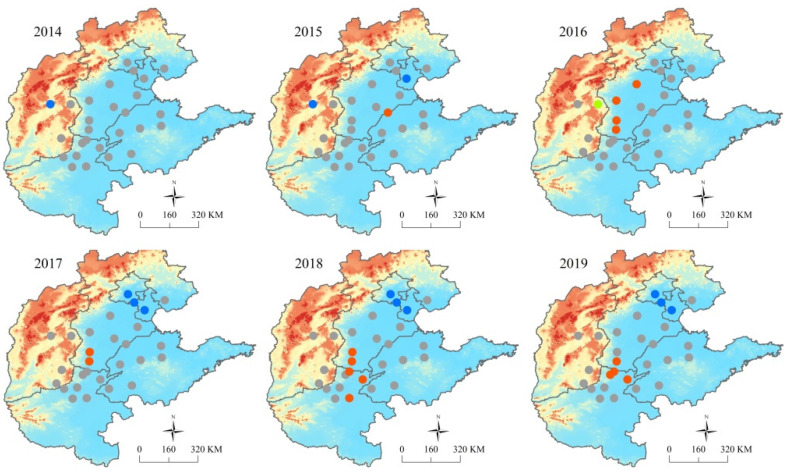

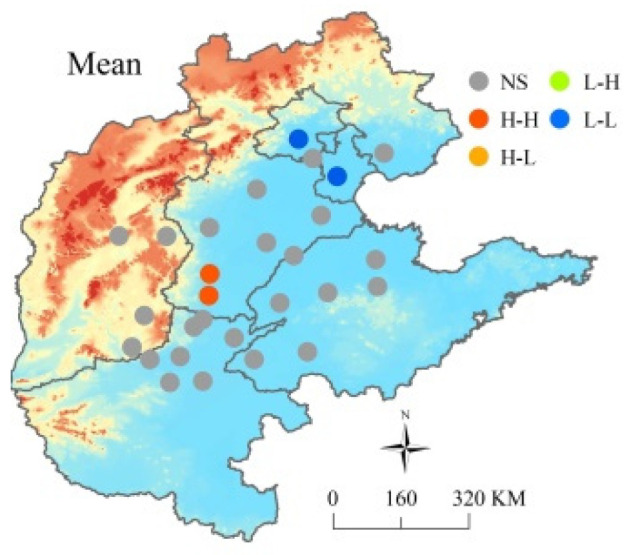
Local spatial autocorrelation features of mean PM_2.5_ concentrations in winter among the 28 air transport channel cities from 2014 to 2019: H–H, city and surrounding cities have high PM_2.5_ concentrations; H–L, city has a high PM_2.5_ concentration, but those of surrounding cities are low; L–H, city has a low PM_2.5_ concentration, but those of surrounding cities are high; L–L, both the city and surrounding cities have low PM_2.5_ concentrations; NS, no significant difference in PM_2.5_ concentration among cities.

Spatial autocorrelation analysis revealed that the average PM_2.5_ concentrations during 2014–2019 had a *Global Moran’s I* of −0.05 (*p* = 0.95) in spring, 0.09 (*p* = 0.35) in summer, 0.20 (*p* = 0.08) in autumn, and 0.30 (*p* = 0.01) in winter, indicating spatial patterns in winter across all channel cities. Local spatial patterns of average PM_2.5_ concentrations in winter displayed clear spatial agglomeration characteristics, with H–H spatial clusters from Dezhou in 2015, to Handan Anyang, Hebi and Puyang in 2019. The L–L spatial clusters appeared in Beijing and Tianjin and persisted thereafter (Figure 3).

#### 3.1.2. Distribution of PM_2.5_/PM_10_ Ratios

**Figure 4:**
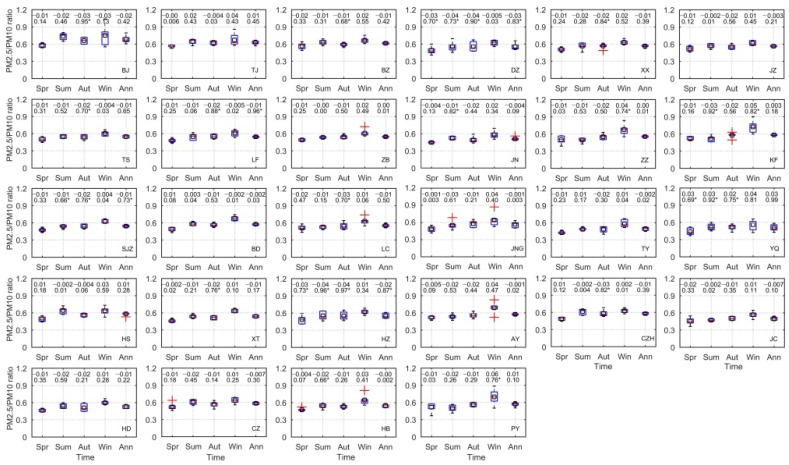
The temporal variation of mean PM_2.5_/PM_10_ ratios in the 28 air transport channel cities during 2014–2019. In each box plot, the bottom and top of the box represent the 25th and 75th percentiles, respectively; horizontal red lines indicate median values; open black squares indicate mean values; whiskers show 1.5 times the interquartile range; and red crosses represent outliers. Linear regression slopes and *R*^2^ values are provided for each city (* *p* < 0.05): BJ, Beijing; TJ, Tianjin; SJZ, Shijiazhuang; TS, Tangshan; LF, Langfang; BD, Baoding; CZ, Cangzhou; HS, Hengshui; XT, Xingtai; HD, Handan; BZ, Binzhou; DZ, Dezhou; ZB, Zibo; JN, Jinan; LC, Liaocheng; JNG, Jining; HZ, Heze; AY, Anyang; HB, Hebi; PY, Puyang; XX, Xinxiang; JZ, Jiaozou; ZZ, Zhengzhou; KF, Kaifeng; TY, Taiyuan; YQ, Yangquan; CHZ, Changzhi; JC, Jincheng.

Annual mean PM_2.5_ concentration accounted for a large fraction of PM_10_ during 2014–2019 in most cities, with a mean PM_2.5_/PM_10_ ratio of 0.56 (Figure 4). PM_2.5_/PM_10_ ratios showed large differences in spatial distribution, with higher ratios found in Beijing (0.68), Tianjin (0.62) and Binzhou (0.61) and lower ratios observed in Taiyuan (0.49), Yangquan (0.51) and Jincheng (0.50), all of which are located in Shanxi Province. By contrast, despite similar spatial distributions of mean PM_2.5_/PM_10_ ratios in each season, clear seasonal differences were observed. Among the 28 cities, 27 cities displayed their highest mean PM_2.5_/PM_10_ ratios in winter (excluding Hengshui), whereas almost all cities showed their lowest mean ratios in spring. From 2014 to 2019, the annual mean PM_2.5_/PM_10_ ratios in most cities of Hebei and Shandong Provinces exhibited a clearly decreasing trend, with significant decreases in Langfang, Shijiazhuang, Dezhou and Heze, whereas most of the other cities fluctuated over time (Figure 4). Compared with spring and summer, mean PM_2.5_/PM_10_ ratios showed particularly large decreases in autumn, with significant reductions in 12 of the 28 cities. By contrast, 23 cites showed a fluctuating upward trend for PM_2.5_/PM_10_ ratios in winter, especially in the cities of Henan Province, such as Puyang (*p* = 0.02), Zhengzhou (*p* = 0.03) and Kaifeng (*p* = 0.01).

**Figure 5:**
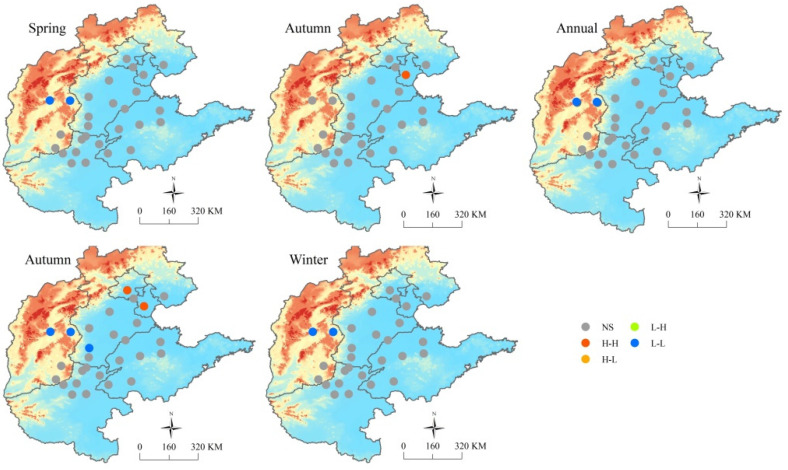
Local spatial autocorrelation features of seasonal and annual mean PM_2.5_/PM_10_ ratios in the 28 air transport channel cities during 2014–2019: H–H, city and surrounding cities have high PM_2.5_ concentrations; H–L, city has a high PM_2.5_ concentration, but those of surrounding cities are low; L–H, city has a low PM_2.5_ concentration, but those of surrounding cities are high; L–L, both the city and surrounding cities have low PM_2.5_ concentrations; NS, no significant difference in PM_2.5_ concentration among cities.

Spatial autocorrelation analysis of mean PM_2.5_/PM_10_ ratios demonstrated that the *Global Moran’s I* ranged from 0.005 to 0.15, with no significant differences at the seasonal or annual scale, indicating that the PM_2.5_/PM_10_ ratio of each city did not influence those of the other cities in aggregate. However, local spatial autocorrelation analysis exhibited L–L spatial clusters of mean PM_2.5_/PM_10_ ratios in Taiyuan and Yangquan during spring and winter and H–H spatial clusters in Tianjin in summer and in both Tianjin and Beijing in autumn (Figure 5).

### 3.2. Relationships between Temporal PM_2.5_ Variation and Meteorological Variables

Partial correlation analysis results for PM_2.5_ concentration with the meteorological variables are shown in Table 1. PM_2.5_ concentration was significantly negatively correlated with *AP*, *T_min_*, *SH* (except in Puyang) and *WS* (except in Yangquan); it was positively correlated with *T_max_* in 23 of the 28 cities and with *H* throughout the study period. By comparison, the correlation between *WD* and PM_2.5_ concentration was positive over the northeastern part of the study area (Beijing, Tianjin, Langfang, Cangzhou, Binzhou, Dezhou, Jinan and Jining), but negative over the southwestern region (Handan, Liaocheng, Heze, Puyang, Xinxiang, Jiaozuo, Kaifeng, Yangquan, Changzhi and Jincheng). Generally, *T_min_* and *H* (especially *T_min_*) were critical factors affecting variation in PM_2.5_ concentration throughout the year.

Seasonal correlations between PM_2.5_ concentration and meteorological variables were generally similar to those at the annual scale, with some exceptions (Table S2). In winter, correlations between PM_2.5_ concentration and *SH* and *H* (especially *H)* were much stronger than those at the annual scale in most cities, whereas those correlations between PM_2.5_ concentration and *T_min_* became slightly weaker. In spring, *T_min_* and *H* were the two most important factors affecting the variation in PM_2.5_ concentration in most cities, whereas the relationship between *AP* and PM_2.5_ concentration was significant only in seven cities. Among the four seasons, PM_2.5_ concentration showed the weakest correlation with AP and H in summer and was more strongly correlated with *T_max_* and *SH* for more cities in summer than in spring and autumn. In autumn, PM_2.5_ concentration was significantly negatively correlated with *T_min_* in 27 cities, with *AP* in 25 cities and with *H* in 24 cities; *T_min_* had the greatest influence on PM_2.5_ concentration variation. Interestingly, partial correlation analysis also showed that PM_2.5_ concentration was positively related to *WD* in winter in eastern cities such as Tianjin, Cangzhou, Xingtai, Binzhou, Dezhou, Zibo, Jinan and Jining, but positively correlated with *WD* in summer in some northern cities (Beijing, Langfang, Baoding, Hengshui, Binzhou, and Taiyuan).

Based on multiple linear regression analysis, meteorological variables account for 31.96% (mean R^2^) of the variation in PM_2.5_ concentration among the 28 cities during the study period; Shijiazhuang had the highest R^2^ value (44.46%), and Jinan had the lowest value (21.30%). At the seasonal scale, the mean R^2^ was highest in winter (0.43), with the highest R^2^ values in Beijing, Tianjin and the cities of Hebei and Shandong Provinces, whereas R^2^ was lowest in summer, with a mean value of 0.13 (Table 2).

### 3.3. Effects of Meteorological Factors on the Spatial Heterogeneity of PM_2.5_

**Figure 6:**
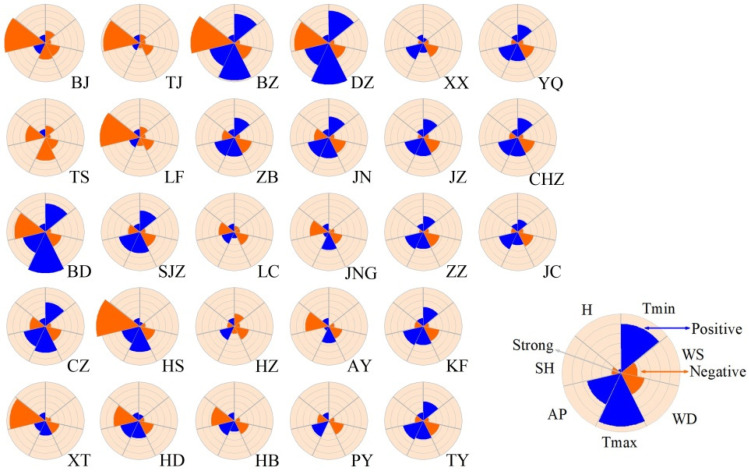
The influence characteristics of meteorological factors for each city based on regression coefficients derived from geographically weighted regression at the annual scale. Blue and orange indicate positive and negative spatial correlations, respectively, between mean PM_2.5_ concentration and meteorological variables. Annuli from the center outward represent the magnitude of the regression coefficient, from lowest to highest: *SH*, sunshine hours; *T_max_*, daily maximum temperature; *T_min_*, daily minimum temperature; *AP*, average atmospheric pressure; *H*, average relative humidity; *WS* maximum wind speed; *WD*, wind direction; BJ, Beijing; TJ, Tianjin; SJZ, Shijiazhuang; TS, Tangshan; LF, Langfang; BD, Baoding; CZ, Cangzhou; HS, Hengshui; XT, Xingtai; HD, Handan; BZ, Binzhou; DZ, Dezhou; ZB, Zibo; JN, Jinan; LC, Liaocheng; JNG, Jining; HZ, Heze; AY, Anyang; HB, Hebi; PY, Puyang; XX, Xinxiang; JZ, Jiaozou; ZZ, Zhengzhou; KF, Kaifeng; TY, Taiyuan; YQ, Yangquan; CHZ, Changzhi; JC, Jincheng.

The geographic distribution of regression coefficient values of seven meteorological factors across the BTH region is shown in Figure 6. The association direction between *SH*, *WS*, *WD* and PM_2.5_ concentration was negative in all cities, especially for *SH* and *WD*, suggesting that increases in *SH* and *WD* had an inhibitory effect on PM_2.5_ concentration. Among the seven meteorological factors examined, *SH* had the strongest influence on PM_2.5_ concentration in Beijing, Tianjin, Langfang, Hengshui, Xingtai, Handan, Binzhou, Liaocheng, Jining, Anyang and Hebi, which are located to the east of the Taihang Mountains. By comparison, *AP* and both temperature factors exhibited a strongly positive effect on PM_2.5_ concentration in the remaining cities. Notably, *T_max_* had the strongest influence in Tangshan, Baoding, Binzhou, Cangzhou and Dezhou, whereas *AP* had the strongest influence in the other 13 cities.

**Figure 7:**
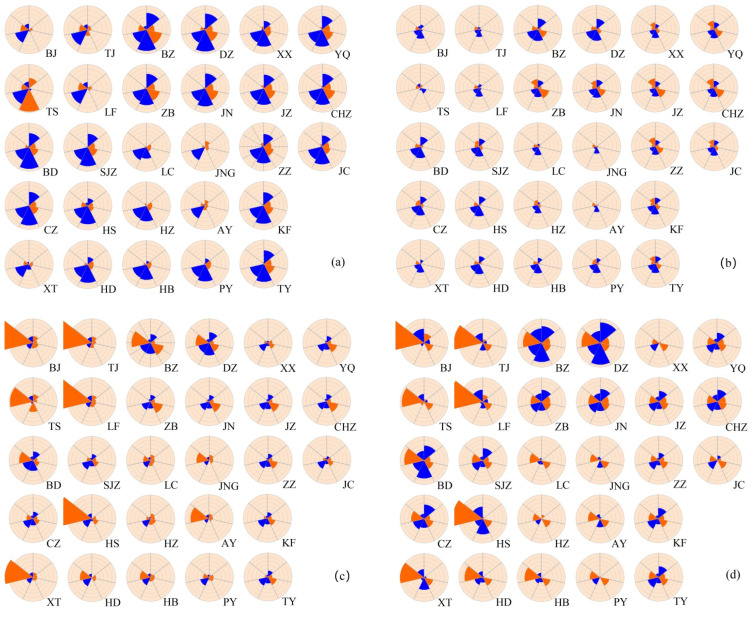
The influence characteristic of meteorological factors for each city based on regression coefficients derived from GWR at the seasonal scale. (**a**) Spring; (**b**) Summer; (**c**) Autumn; (**d**) Winter. Blue and orange indicate positive and negative spatial correlations, respectively, between mean PM_2.5_ concentration and meteorological variables. Annuli from the center outward represent the magnitude of the regression coefficient, from lowest to highest. See Figure 7 for legend: BJ, Beijing; TJ, Tianjin; SJZ, Shijiazhuang; TS, Tangshan; LF, Langfang; BD, Baoding; CZ, Cangzhou; HS, Hengshui; XT, Xingtai; HD, Handan; BZ, Binzhou; DZ, Dezhou; ZB, Zibo; JN, Jinan; LC, Liaocheng; JNG, Jining; HZ, Heze; AY, Anyang; HB, Hebi; PY, Puyang; XX, Xinxiang; JZ, Jiaozou; ZZ, Zhengzhou; KF, Kaifeng; TY, Taiyuan; YQ, Yangquan; CHZ, Changzhi; JC, Jincheng.

Significant spatial heterogeneity was detected in both the direction and strength of the environmental factors among different seasons based on the GWR method (Figure 7). In spring, *AP* and *T_max_* had the greatest influence on the spatial distribution of PM_2.5_ concentration. *AP* had a significant positive relationship with PM_2.5_ concentration in Beijing, Tianjin, Langfang, Xingtai, Liaocheng, Jining and Anyang, whereas *T_max_* had the largest positive effect on PM_2.5_ concentration in the remaining cities (about 71.43% of all cities). *WS* and *WD* were negatively correlated with PM_2.5_ concentration in all cities except Tangshan, but had a smaller impact on PM_2.5_ concentration in Beijing, Tianjin, Langfang, Hengshui, Xingtai, Handan, Liaocheng, Jining, Heze, Anyang, Hebi and Puyang (Figure 7a). In summer, the impact patterns of temperature, wind and atmospheric pressure were generally similar to those in spring, but to a much smaller degree. *H* displayed a clear negative relationship with PM_2.5_ concentration in most cities of Shanxi, Henan and Shandong Provinces (Figure 7b), indicating that higher humidity was associated with lower PM_2.5_ concentration.

In autumn, the relationships between *SH*, *WS* and *WD* and PM_2.5_ concentration were negative, whereas *AP* and *H* were positively related with PM_2.5_ concentration in all cities. These patterns were comparable with those at the annual scale. In Beijing, Tianjin, Tangshan, Langfang, Baoding, Hengshui, Xingtai, Handan, Binzhou, Dezhou, Jining, Anyang and Hebi, *SH* played the most dominant role in determining PM_2.5_ concentration, whereas *AP* was the dominant variable in most other cities, which were mainly located in the southern part of the BTH region (about 50% of all cities). Correlations between meteorological factors and PM_2.5_ concentration were generally similar in winter and autumn (Figure 7c,d), but with some differences. For instance, *SH* remained the strongest influence in 13 cities, whereas its impact weakened in nine cities and strengthened in most central and southern cities. *AP* exerted an increasing impact in southern cities and maintained a dominant role in Jiaozuo, Zhengzhou, Kaifeng, Taiyuan, Yangquan and Jincheng. *H* showed a negative relationship with PM_2.5_ concentration in Heze and Xinxiang, and an enhanced positive relationship in 22 cities. Temperature had the largest positive effect on PM_2.5_ concentration in eastern and central cities, including Shijiazhuang, Cangzhou, Binzhou, Dezhou, Zibo, Jinan and Changzhi (Figure 7d).

## 4. Discussion

### 4.1. Spatiotemporal Variations in PM_2.5_

In this study, we found that annual mean PM_2.5_ concentration decreased in the 28 channel cities (significantly in 24 cities) in the BTH region during 2014–2019. Similar trends have been reported in previous studies. For example, average annual PM_2.5_ concentration was reported to have decreased throughout the BTH region during 2013–2016 [46] and 2013–2018 [32,33] and to have declined by about 40% in Beijing from 2013–2018 [18,36]. These decreases in PM_2.5_ concentration can be attributed mainly to the Action Plan on Prevention and Control of Air Pollution in 2013 and the Air Pollution Prevention and Control Program in Beijing, Tianjin, Hebei and surrounding areas in 2017. However, it was noteworthy that the rate of decline in PM_2.5_ concentration slowed dramatically after 2017, even fluctuating upward in some cities (Figure S1). We also found that the annual mean PM_2.5_ concentration of all channel cities in the BTH region remained much higher than the Grade II standard of CAAQS in 2019, which indicates that further measures are still strongly needed despite the improvements attributed to control strategies implemented within the past decade.

The results of this study demonstrate that PM_2.5_ concentration exhibits a distinct seasonal cycle, with less severe PM_2.5_ pollution in summer and greater pollution in winter, probably due to the combined impact of unfavorable weather conditions and coal and biomass combustion for residential heating [32,47]. The PM_2.5_/PM_10_ ratio also showed a seasonal variation, decreasing in spring and peaking in winter, which can be attributed to increasing fuel consumption [48] and the secondary formation of PM_2.5_ in winter [23]. In addition, our results showed significant reductions in PM_2.5_ concentration in only four cities in winter, the most polluted season, whereas PM_2.5_/PM_10_ ratios of most cites showed a fluctuating upward trend. The increase in PM_2.5_/PM_10_ ratios also have been reported at several monitoring sites in the UK [49] and India [50]. These results indicate that more control measures should focus on PM_2.5_ emissions and their formation mechanisms in winter.

Air pollution has been suggested to be strongly affected by pollutants from adjacent cities [51]. The intensity of spatially dependent air pollution tends to differ seasonally due to differences in synoptic patterns and emission characteristics [28]. For example, Sun et al. (2019) reported that spatial agglomeration of air pollution was stronger in winter and weaker in summer [51]. Using a pollutant tracing model, Wang et al. (2015) explored the regional contributions of PM_2.5_ pollution in Shijiazhuang, Xingtai and Handan, and found that regional contributions were larger in January than in July [52]. In accordance with these findings, our results showed clear large scale spatial autocorrelation of PM_2.5_ concentration occurred in autumn and winter, indicating that PM_2.5_ pollution in adjacent cities may strongly interact among the 28 cities of the BTH region during the latter half of the year. However, the local spatial autocorrelation results of our study showed that regions with high agglomeration of PM_2.5_ concentration were mainly concentrated in the southern Hebei Province and northern Henan Province, but with some spatial variation, suggesting that joint variable control strategies should be emphasized for PM_2.5_ pollution over time.

### 4.2. Relationship between PM_2.5_ Concentration and Meteorological Factors

Meteorological conditions played an important role in influencing PM_2.5_ concentration. In this study, *T_min_* and *H* were the leading factors affecting PM_2.5_ concentration at the annual scale. *T_min_* was negatively correlated with PM_2.5_ concentration, which is consistent with the fact that air quality is better in summer and worse in winter. This negative correlation may be closely related to less dispersion and dilution of air pollutants at lower *T_min_*. At low minimum temperature, air convection is weak and temperature inversion can easily occur. These conditions are not conducive to the diffusion and dilution of air pollutants [33]. By contrast, *H* had the strongest positive influence on PM_2.5_ concentration. Increases in *H* increase the production of sulfate aerosols via aqueous reactions and also significantly affect the partitioning of HNO_3_ between the gas and particulate phases, leading to enhanced nitrate formation [34]. In addition, high humidity conditions promote the production of secondary organic aerosols [53]. For example, the contribution of more-oxidized secondary organic aerosols to organic aerosols increases substantially as a function of relative humidity [54]. However, some studies have reported that particulate pollutants tend to gather mass and fall to the ground on days with high relative humidity [55]. This divergence may be attributed partly to the degree of relative humidity. At moderate relative humidity, particulate pollutants tend to cluster, and environmental quality worsens. However, as humidity increases, these particles are scavenged through precipitation [56,57].

The correlations between meteorological factors and PM_2.5_ concentration showed some interesting characteristics in different seasons. Generally, meteorological factors had a greater influence on PM_2.5_ concentration in all cities in winter, and the effects of these factors were much stronger in winter than in other seasons, which is consistent with the findings of a previous study [37]. In winter, unfavorable circulation conditions, such as the positive humidity anomalies, a stable boundary layer and air stagnation occur frequently in east Asian monsoonal climates, exacerbating air pollution [33]. Fewer meteorological factors influenced PM_2.5_ concentration in summer, when the variation in PM_2.5_ concentration depended mainly on changes in *T_max_* and *SH*. Previous studies have reported that increases in temperature increased radical production rates and promoted aerosol formation, but the atmospheric photolysis occurred on organic carbon, reducing PM_2.5_ concentration [37,40] (Chen et al., 2017; Dawson et al., 2007). These findings are consistent with our results to some extent. However, other studies reported that high temperatures caused atmospheric convection, resulting in greater dispersion and dilution of air pollutants, and photochemical reactions of precursor volatile organic compounds under sunlight could result in secondary organic aerosols and photochemical smog [58,59]. Therefore, further studies are needed to explore the influence mechanisms of *T_max_* and *SH* on PM_2.5_ concentration in this region.

The spatial distribution of air pollution has been associated with meteorological factors in previous studies [55]. Liu et al. (2017) found that temperature had a significant positive effect on air pollution distribution, whereas wind speed and precipitation had significant negative effects in 289 Chinese cities in 2014 [42]. Sun et al., 2019, found that temperature and wind speed were significantly negatively correlated with the urban air quality index in 338 Chinese cities [51]. In contrast to some previous studies, our GWR results demonstrated that *WS* was negatively correlated with PM_2.5_ concentration in all cities, while *T_max_* and *T_min_* exhibited a positive correlation with PM_2.5_ concentration in 82.14% and 60.71% of all cities, respectively, at the annual scale. The influence of temperature seems complex and differs regionally. We also found that *SH* strongly affected the PM_2.5_ concentration in areas east of the Taihang mountains, whereas *AP* strongly affected the PM_2.5_ concentration in cities west of this mountain range. These differences can be attributed partly to the topographical characteristics. Unique atmospheric circulation subject to the particular topography has been suggested to develop spatial distribution characteristics of air pollutants [60,61].

This study had some limitations. First, PM_10_ and PM_2.5_ concentration data for Jincheng was collected and analyzed from 1 March 2015 due to the limited monitoring record. In addition, there were about 44 missing air pollution records among 28 cities and few zero values for PM_10_ concentration, which were removed to calculate the PM_2.5_/PM_10_ ratios. Although we applied interpolation to maintain the continuity and comparability of these time series data, some uncertainty remains. Moreover, although the Local Moran’s I method used in this study could indeed shed light on the potential interdependence and specific spatial agglomeration patterns of PM_2.5_ concentration, the limited observations for 28 cities were slightly less than the restriction on the numbers required by this method. Additionally, the temporal and spatial variations of PM_2.5_ concentration were strongly correlated with meteorological factors, and their relationships varied significantly across seasons and geographical locations, probably relating to PM_2.5_ components. To better address this problem, GWR was applied in this study to capture the spatial nonstationary characteristics, but it could not simultaneously deal with temporal nonstationarity [62,63], which also could result in some uncertainty. Future research is required to elucidate these mechanisms.

## 5. Conclusions

In this study, we investigated the spatial and temporal variation of PM_2.5_ concentration in 28 cities within the atmospheric pollution transmission channel in the BTH region with associated meteorological influences during 2014–2019. Average PM_2.5_ concentration appeared to decrease significantly in most cities at the annual scale, but exhibited clear seasonal and spatial variation. The mean PM_2.5_ concentration demonstrated a significant decreasing trend in more than 20 cities in spring and summer, but only 4 cities in winter, indicating that strict measures should be continued, especially in the most severely polluted season, despite successful air pollution control plans implemented within the past decade. Temporal variation in daily PM_2.5_ concentration was strongly correlated with minimum temperature and average relative humidity. The higher the PM_2.5_ temporal concentration, the stronger the influence of the meteorological factors, with a mean explanatory power of 31.96% for the 28 cities. Spatially, wind speed was negatively correlated with PM_2.5_ concentration for all cities, while maximum temperature and minimum temperature exhibited a positive correlation with PM2.5 concentration in 82.14% and 60.71% cities, respectively. These results will provide valuable references for understanding the spatiotemporal variation of PM_2.5_ pollution, as well as for future clean air policy making.

## Figures and Tables

**Table 1:** Partial correlations of PM_2.5_ concentration with meteorological variables based on daily data during 2014–2019: *SH*, sunshine hours; *T_max_*, daily maximum temperature; *T_min_*, daily minimum temperature; *AP*, average atmospheric pressure; *H*, average relative humidity; *WS* maximum wind speed; *WD*, wind direction; BJ, Beijing; TJ, Tianjin; SJZ, Shijiazhuang; TS, Tangshan; LF, Langfang; BD, Baoding; CZ, Cangzhou; HS, Hengshui; XT, Xingtai; HD, Handan; BZ, Binzhou; DZ, Dezhou; ZB, Zibo; JN, Jinan; LC, Liaocheng; JNG, Jining; HZ, Heze; AY, Anyang; HB, Hebi; PY, Puyang; XX, Xinxiang; JZ, Jiaozou; ZZ, Zhengzhou; KF, Kaifeng; TY, Taiyuan; YQ, Yangquan; CHZ, Changzhi; JC, Jincheng.

Variable	Correlation Coefficient																											
	BJ	TJ	TS	LF	BD	SJZ	CZ	HS	XT	HD	BZ	DZ	ZB	JN	LC	JNG	HZ	AY	HB	PY	XX	JZ	ZZ	KF	TY	YQ	CHZ	JC
*SH*	**−0.15**	**−0.20**	**−0.20**	**−0.16**	**−0.28**	**−0.25**	**−0.10**	**−0.18**	**−0.25**	**−0.13**	**−0.31**	**−0.17**	**−0.29**	**−0.13**	**−0.08**	**−0.11**	**−0.13**	**−0.18**	**−0.14**	0.01	**−0.12**	**−0.06**	**−0.10**	**−0.08**	**−0.21**	**−0.21**	**−0.07**	**−0.05**
*T_max_*	0.02	**0.21**	**0.18**	**0.12**	**0.09**	**0.16**	**0.04**	**0.14**	**0.20**	**0.07**	**0.28**	**0.13**	**0.23**	0.02	**0.06**	**0.13**	**0.11**	**0.10**	−0.03	**−0.05**	0.03	0.01	0.00	**0.08**	**0.18**	**0.16**	**0.06**	**0.14**
*T_min_*	**−0.24**	**−0.38**	**−0.33**	**−0.31**	**−0.37**	**−0.39**	**−0.22**	**−0.34**	**−0.32**	**−0.31**	**−0.39**	**−0.28**	**−0.38**	**−0.12**	**−0.30**	**−0.29**	**−0.28**	**−0.29**	**−0.21**	**−0.27**	**−0.22**	**−0.23**	**−0.25**	**−0.25**	**−0.37**	**−0.33**	**−0.19**	**−0.36**
*AP*	**−0.24**	**−0.20**	**−0.18**	**−0.20**	**−0.23**	**−0.26**	**−0.17**	**−0.17**	**−0.12**	**−0.22**	**−0.15**	**−0.14**	**−0.13**	**−0.08**	**−0.15**	**−0.12**	**−0.10**	**−0.19**	**−0.17**	**−0.16**	**−0.17**	**−0.22**	**−0.17**	**−0.12**	**−0.19**	**−0.17**	**−0.13**	**−0.13**
*H*	**0.28**	**0.28**	**0.28**	**0.28**	**0.18**	**0.20**	**0.22**	**0.27**	**0.17**	**0.26**	**0.09**	**0.20**	**0.20**	**0.09**	**0.29**	**0.19**	**0.18**	**0.12**	**0.21**	**0.26**	**0.18**	**0.21**	**0.13**	**0.16**	**0.26**	**0.20**	**0.09**	**0.20**
*WS*	**−0.09**	**−0.28**	**−0.12**	**−0.12**	**−0.19**	**−0.26**	**−0.11**	**−0.15**	**−0.25**	**−0.16**	**−0.26**	**−0.14**	**−0.12**	**−0.20**	**−0.18**	**−0.12**	**−0.26**	**−0.17**	**−0.11**	**−0.20**	**−0.16**	**−0.13**	**−0.23**	**−0.22**	**−0.13**	−0.03	**−0.24**	**−0.19**
*WD*	**0.05**	**0.05**	−0.04	**0.08**	−0.03	−0.04	**0.11**	0.04	0.01	**−0.07**	**0.09**	**0.11**	0.02	**0.08**	**−0.05**	**0.05**	**−0.05**	−0.01	−0.01	**−0.05**	**−0.06**	**−0.06**	−0.03	**−0.05**	0.02	**−0.15**	**−0.12**	**−0.10**

Note: Bold font denotes *p* < 0.05.

**Table 2:** Coefficients of determination (R^2^) for multiple linear regression of PM_2.5_ concentration with meteorological variables based on daily data from 2014 to 2019 at the annual and seasonal scales: BJ, Beijing; TJ, Tianjin; SJZ, Shijiazhuang; TS, Tangshan; LF, Langfang; BD, Baoding; CZ, Cangzhou; HS, Hengshui; XT, Xingtai; HD, Handan; BZ, Binzhou; DZ, Dezhou; ZB, Zibo; JN, Jinan; LC, Liaocheng; JNG, Jining; HZ, Heze; AY, Anyang; HB, Hebi; PY, Puyang; XX, Xinxiang; JZ, Jiaozou; ZZ, Zhengzhou; KF, Kaifeng; TY, Taiyuan; YQ, Yangquan; CHZ, Changzhi; JC, Jincheng.

Period	Coefficient of Determination																											
	BJ	TJ	TS	LF	BD	SJZ	CZ	HS	XT	HD	BZ	DZ	ZB	JN	LC	JNI	HZ	AY	HB	PY	XX	JZ	ZZ	KF	TY	YQ	CHZ	JC
Spring	**0.30**	**0.31**	**0.26**	**0.27**	**0.28**	**0.39**	**0.16**	**0.27**	**0.30**	**0.24**	**0.24**	**0.13**	**0.21**	**0.14**	**0.19**	**0.09**	**0.21**	**0.17**	**0.17**	**0.23**	**0.19**	**0.26**	**0.19**	**0.12**	**0.28**	**0.25**	**0.16**	**0.16**
Summer	**0.29**	**0.16**	**0.04**	**0.09**	**0.15**	**0.31**	**0.06**	**0.11**	**0.19**	**0.04**	**0.23**	**0.07**	**0.19**	**0.10**	**0.19**	**0.08**	**0.19**	**0.08**	**0.12**	**0.07**	**0.06**	**0.05**	**0.06**	**0.07**	**0.19**	**0.16**	**0.07**	**0.06**
Autumn	**0.36**	**0.43**	**0.39**	**0.30**	**0.34**	**0.41**	**0.20**	**0.31**	**0.34**	**0.20**	**0.22**	**0.24**	**0.22**	**0.13**	**0.26**	**0.16**	**0.18**	**0.21**	**0.24**	**0.18**	**0.19**	**0.19**	**0.19**	**0.14**	**0.33**	**0.21**	**0.16**	**0.23**
Winter	**0.52**	**0.53**	**0.56**	**0.57**	**0.45**	**0.54**	**0.47**	**0.41**	**0.40**	**0.45**	**0.47**	**0.44**	**0.47**	**0.44**	**0.42**	**0.35**	**0.39**	**0.37**	**0.38**	**0.36**	**0.33**	**0.39**	**0.39**	**0.36**	**0.45**	**0.51**	**0.30**	**0.39**
Year	**0.30**	**0.38**	**0.31**	**0.35**	**0.40**	**0.44**	**0.26**	**0.36**	**0.35**	**0.34**	**0.30**	**0.28**	**0.31**	**0.21**	**0.35**	**0.29**	**0.35**	**0.31**	**0.32**	**0.35**	**0.29**	**0.33**	**0.33**	**0.31**	**0.32**	**0.28**	**0.24**	**0.31**

Note: Bold font denotes *p* < 0.05.

## Data Availability

The datasets used and analyzed during the current study are available from the author on reasonable request.

## Supplementary Materials

The following supporting information can be downloaded at: https://www.mdpi.com/article/10.3390/ijerph19031607/s1, Figure S1: The cities with PM_2.5_ concentration fluctuating upward after 2017; Table S1: Summary of major air pollution control measures taken in <The work plan for air pollution prevention and control in Beijing, Tianjin, Hebei and surrounding areas in 2017>; Table S2: Partial correlations of PM_2.5_ concentration with meteorological variables at the seasonal scale based on daily data during 2014–2019.
