# Supplementary material for: Meteorological Influences on Spatiotemporal Variation of PM2.5 Concentrations in Atmospheric Pollution Transmission Channel Cities of the Beijing–Tianjin–Hebei Region, China
Source: Int J Environ Res Public Health. 2022 Jan 30;19(3):1607. doi: 10.3390/ijerph19031607 (PMC8834796; doi:10.3390/ijerph19031607)
Supplement: Supplementary file 1 [file ijerph-19-01607-s001.zip › ijerph-1523268-supplementary.pdf]

Table S1 Summary of major air pollution control measures taken in《The work plan for air pollution prevention and control in Beijing, Tianjin, Hebei and surrounding areas in 2017》

Number	Main tasks	Main measures	Major Specific indicators	Scope
1	Industrial structure adjustment	Increase the capacity to resolve overcapacity	Resolve steel overcapacity	28 cities in atmospheric pollution transmission channel of the Beijing–Tianjin–Hebei region, China
		ban on illegal "small scattered pollution" enterprises	Below pollutant discharge standard<br>Incomplete procedures	
2	Comprehensively promote clean heating in winter	Implement key projects of clean heating in winter	50−100 thousand households replacing coal with gas or electricity per city<br>Construction of "no coal zone"	
		Complete the "clearing" of small coal−fired boilers	Eliminate coal−fired boilers of 10 steam tons and below	
		Achieve negative growth in total coal consumption	Achieved full coverage of central heating or clean energy heating for county with a population of more than 200 thousand	
3	Comprehensive control of industrial air pollution	Implementation of special emission limits	Special emission limits for steel and coal−fired boilers	
		Comprehensively promote the management of pollutant discharge permits	Pollutant discharge permits for steel, power plant and cement industries	
		Comprehensive control of volatile organic compounds (VOCs)		
4	Implement staggered peak production in heating season	Fully implement staggered peak production for cement, casting and other industries	Staggered peak production for cement, casting and other industries<br>Shut down coal fired generator without ultra−low emission	
		Production restrictions for iron and steel enterprises in key cities	Steel production capacity limiting 50% in heating season in key cities	
		Production regulation of electrolytic aluminum and chemical enterprises	Electrolytic aluminum, alumina production capacity limiting 30%,<br>Carbon enterprise production capacity limiting 50% in heating season	
5	Strictly control motor vehicle emissions	Ban on accepting coal transportation by road in Tianjin Port		
		Comprehensively strengthen the monitoring capacity of motor vehicle emission	Screening diesel trucks and high emission gasoline vehicles	
		Strengthen diesel vehicle management and control	Heavy diesel vehicles control in Sixth Ring Road in Beijing	
		Strengthen the supervision and management of oil quality and vehicle urea	Supplying with gasoline and diesel meeting the national six standards<br>No selling ordinary diesel	
6	Improve the level of urban management	Strictly control dust emission	Dustfall less than 9 tons/month/square kilometers in Beijing, Langfang and Baoding	
		Fully implement the requirements of no burning and no emission	No open burning<br>Restriction of fireworks and firecrackers	

Table S2. Partial correlations of $PM_{2.5}$ concentration with meteorological variables at the seasonal scale based on daily data during 2014–2019.

Scale	Var	Correlation coefficient																											
		BJ	TJ	TS	LF	BD	SJZ	CZ	HS	XT	HD	BZ	DZ	ZB	JN	LC	JNI	HZ	AY	HB	PY	XX	JZ	ZZ	KF	TY	YQ	CZ	JC
Spring	*SH*	−0.06	−0.21	−0.12	−0.11	−0.20	−0.18	0.00	−0.17	−0.23	−0.06	−0.28	−0.05	−0.29	−0.08	−0.05	−0.16	−0.03	−0.17	−0.13	−0.04	−0.06	0.01	−0.02	0.01	−0.29	−0.17	−0.17	0.04
	T_{max}	−0.01	0.27	0.20	0.12	0.14	0.19	0.09	0.20	0.29	0.13	0.32	0.10	0.29	0.02	0.06	0.12	0.01	0.16	0.08	0.08	0.03	0.01	0.02	0.02	0.27	0.18	0.19	0.09
	T_{min}	−0.19	−0.32	−0.25	−0.25	−0.32	−0.35	−0.12	−0.33	−0.30	−0.26	−0.31	−0.17	−0.31	0.00	−0.17	−0.12	−0.08	−0.29	−0.20	−0.23	−0.15	−0.16	−0.14	−0.08	−0.36	−0.28	−0.25	−0.27
	AP	−0.18	−0.06	−0.05	−0.09	−0.09	−0.18	0.00	−0.05	0.15	−0.03	0.02	−0.03	0.02	0.04	0.02	0.02	0.00	−0.06	−0.03	−0.02	−0.04	−0.06	−0.03	0.01	−0.15	−0.09	−0.04	−0.07
	H	0.32	0.25	0.32	0.33	0.28	0.21	0.32	0.30	0.23	0.38	0.06	0.24	0.14	0.13	0.28	0.14	0.17	0.15	0.26	0.33	0.25	0.32	0.22	0.20	0.21	0.23	0.14	0.22
	WS	−0.15	−0.30	−0.13	−0.05	−0.13	−0.36	−0.11	−0.14	−0.25	−0.05	−0.28	−0.09	−0.13	−0.24	−0.20	−0.06	−0.34	−0.09	−0.04	−0.19	−0.12	−0.10	−0.21	−0.19	−0.14	0.08	−0.17	−0.14
	WD	0.02	−0.02	−0.06	0.08	−0.10	−0.12	0.08	0.09	−0.02	0.06	0.14	0.07	−0.01	0.04	−0.06	0.04	−0.04	0.04	−0.02	−0.09	−0.01	−0.06	−0.01	0.05	0.05	−0.25	−0.08	−0.04
Summer	*SH*	−0.27	−0.32	−0.10	−0.09	−0.28	−0.44	−0.08	−0.20	−0.33	−0.10	−0.35	−0.20	−0.38	−0.21	−0.17	−0.21	−0.24	−0.22	−0.33	−0.16	−0.15	−0.03	−0.19	−0.19	−0.26	−0.15	−0.02	−0.10
	T_{max}	0.12	0.26	0.05	0.07	0.18	0.26	0.04	0.17	0.27	0.10	0.22	0.12	0.35	0.15	0.29	0.13	0.22	0.18	0.20	0.14	0.09	−0.05	0.15	0.18	0.27	0.13	0.21	0.20
	T_{min}	0.11	−0.10	0.07	0.09	0.02	−0.19	0.13	−0.03	−0.12	−0.10	−0.09	0.03	−0.34	−0.17	−0.27	−0.18	−0.22	−0.12	−0.16	−0.14	−0.15	−0.08	−0.18	−0.15	−0.02	0.10	−0.17	−0.15
	AP	0.04	0.01	−0.03	0.01	−0.01	−0.19	0.05	0.10	0.09	−0.02	0.00	0.10	0.02	0.07	0.11	0.06	0.05	0.05	−0.03	0.06	0.00	−0.13	0.03	0.07	−0.03	0.03	0.06	0.01
	H	0.09	−0.03	−0.05	0.05	−0.02	−0.04	−0.07	0.11	0.04	0.09	−0.20	−0.09	0.04	−0.06	0.22	−0.12	−0.07	−0.04	0.02	−0.03	−0.02	−0.01	−0.07	−0.09	0.05	0.07	0.18	0.09
	WS	0.11	−0.11	0.01	−0.04	−0.18	−0.33	−0.03	−0.10	−0.14	−0.13	−0.24	−0.10	−0.10	−0.15	−0.21	0.02	−0.35	−0.10	−0.10	−0.18	−0.14	−0.08	−0.11	−0.12	−0.01	0.08	−0.11	−0.16
	WD	0.30	0.05	0.07	0.11	0.12	−0.06	0.04	0.14	0.07	−0.01	0.11	0.01	0.00	0.11	0.02	0.02	−0.08	0.08	−0.01	0.02	0.01	−0.10	−0.01	0.03	0.13	−0.19	−0.01	−0.06
Autumn	*SH*	−0.08	−0.18	−0.22	−0.17	−0.33	−0.29	−0.11	−0.21	−0.28	−0.17	−0.25	−0.29	−0.26	−0.07	−0.05	−0.17	−0.11	−0.22	−0.16	0.02	−0.14	−0.04	−0.11	−0.07	−0.20	−0.22	−0.07	−0.06
	T_{max}	−0.10	0.10	0.09	0.06	0.09	0.16	−0.03	0.16	0.27	0.10	0.27	0.18	0.26	−0.03	0.10	0.23	0.11	0.14	0.03	−0.06	0.09	0.04	0.02	0.08	0.19	0.18	0.05	0.18
	T_{min}	−0.10	−0.26	−0.24	−0.21	−0.32	−0.36	−0.13	−0.32	−0.35	−0.25	−0.32	−0.29	−0.37	−0.03	−0.25	−0.33	−0.22	−0.30	−0.25	−0.21	−0.22	−0.19	−0.21	−0.21	−0.42	−0.34	−0.17	−0.36
	AP	−0.31	−0.26	−0.19	−0.21	−0.28	−0.33	−0.17	−0.19	−0.10	−0.18	−0.10	−0.17	−0.10	−0.07	−0.08	−0.11	−0.06	−0.21	−0.15	−0.13	−0.12	−0.15	−0.15	−0.11	−0.30	−0.24	−0.17	−0.14
	H	0.27	0.32	0.33	0.19	0.12	0.11	0.17	0.27	0.17	0.22	0.15	0.13	0.22	0.11	0.32	0.16	0.14	0.05	0.16	0.15	0.20	0.17	0.04	0.06	0.29	0.15	−0.02	0.13
	WS	−0.17	−0.33	−0.16	−0.13	−0.17	−0.23	−0.11	−0.15	−0.28	−0.12	−0.17	−0.14	−0.06	−0.19	−0.13	−0.04	−0.24	−0.17	−0.04	−0.21	−0.14	−0.13	−0.22	−0.25	−0.09	0.04	−0.25	−0.17
	WD	0.02	0.09	0.02	0.11	0.00	−0.08	0.09	0.05	−0.05	−0.02	0.10	0.06	0.04	0.02	−0.02	0.01	−0.08	−0.03	0.05	0.06	−0.04	−0.02	−0.03	−0.07	0.09	−0.11	−0.13	−0.09
Winter	*SH*	−0.16	−0.22	−0.34	−0.26	−0.38	−0.39	−0.26	−0.31	−0.32	−0.30	−0.36	−0.27	−0.39	−0.31	−0.17	−0.13	−0.25	−0.28	−0.21	−0.17	−0.26	−0.19	−0.18	−0.21	−0.26	−0.38	−0.17	−0.16
	T_{max}	0.01	0.25	0.26	0.24	0.27	0.31	0.28	0.29	0.25	0.31	0.39	0.30	0.29	0.25	0.20	0.24	0.29	0.22	0.10	0.15	0.15	0.14	0.12	0.24	0.24	0.30	0.13	0.29
	T_{min}	−0.08	−0.23	−0.24	−0.27	−0.31	−0.34	−0.21	−0.25	−0.20	−0.28	−0.27	−0.19	−0.20	−0.09	−0.17	−0.25	−0.20	−0.07	0.02	−0.10	−0.11	−0.15	−0.07	−0.19	−0.19	−0.14	0.01	−0.22
	AP	−0.22	−0.16	−0.14	−0.14	−0.14	−0.18	−0.16	−0.07	−0.17	−0.10	−0.13	−0.07	−0.12	−0.02	−0.10	−0.07	−0.04	−0.04	−0.05	−0.03	−0.06	−0.10	−0.07	−0.03	−0.05	−0.02	0.01	0.06
	H	0.46	0.37	0.40	0.44	0.27	0.35	0.35	0.30	0.28	0.40	0.23	0.32	0.37	0.30	0.36	0.38	0.35	0.28	0.37	0.33	0.25	0.31	0.26	0.29	0.45	0.39	0.20	0.34
	WS	−0.04	−0.26	−0.07	−0.07	−0.14	−0.23	−0.16	−0.12	−0.18	−0.20	−0.24	−0.22	−0.24	−0.31	−0.24	−0.12	−0.31	−0.22	−0.18	−0.27	−0.18	−0.16	−0.21	−0.24	−0.09	−0.08	−0.29	−0.08
	WD	−0.02	0.09	0.04	0.04	0.05	−0.02	0.19	0.01	0.09	−0.21	0.13	0.28	0.13	0.13	−0.11	0.13	−0.17	0.02	−0.03	−0.08	−0.05	0.03	0.00	−0.09	−0.05	−0.08	−0.13	−0.13

Note：Red font denotes $P<0.05$.

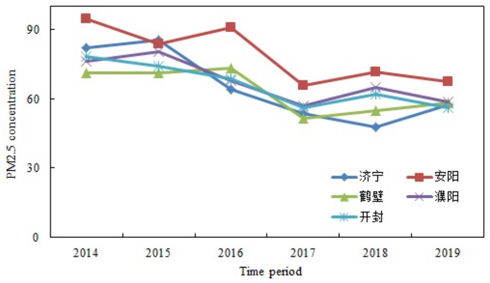

Figure S1 The cities with $PM_{2.5}$ concentration fluctuating upward after 2017.
